# Rare variants of the 3’-5’ DNA exonuclease *TREX1* in early onset small vessel stroke

**DOI:** 10.12688/wellcomeopenres.12631.1

**Published:** 2017-11-02

**Authors:** Sarah McGlasson, Kristiina Rannikmäe, Steven Bevan, Clare Logan, Louise S. Bicknell, Alexa Jury, UK Young Lacunar Stroke Study, Andrew P. Jackson, Hugh S. Markus, Cathie Sudlow, David P.J. Hunt

**Affiliations:** 1 Centre for Clinical Brain Sciences, University of Edinburgh, Edinburgh, EH16 4SB, UK; 2 MRC Human Genetics Unit, Institute of Genetics and Molecular Medicine, University of Edinburgh, Edinburgh, EH4 2XU, UK; 3 Stroke Research Group, Department of Clinical Neurosciences, Cambridge University, Cambridge, CB2 2PY , UK; 4 Joseph Banks Laboratories, University of Lincoln, Lincoln, LN6 7DL, UK

**Keywords:** TREX1, neuroinflammation, small vessel stroke, lacunar stroke

## Abstract

***Background:*** Monoallelic and biallelic mutations in the exonuclease *TREX1* cause monogenic small vessel diseases (SVD). Given recent evidence for genetic and pathophysiological overlap between monogenic and polygenic forms of SVD, evaluation of *TREX1* in small vessel stroke is warranted.

***Methods:*** We sequenced the *TREX1* gene in an exploratory cohort of patients with lacunar stroke (Edinburgh Stroke Study, n=290 lacunar stroke cases). We subsequently performed a fully blinded case-control study of early onset MRI-confirmed small vessel stroke within the UK Young Lacunar Stroke Resource (990 cases, 939 controls).

***Results:*** No patients with canonical disease-causing mutations of *TREX1* were identified in cases or controls. Analysis of an exploratory cohort identified a potential association between rare variants of *TREX1* and patients with lacunar stroke. However, subsequent controlled and blinded evaluation of *TREX1* in a larger and MRI-confirmed patient cohort, the UK Young Lacunar Stroke Resource, identified heterozygous rare variants in 2.1% of cases and 2.3% of controls. No association was observed with stroke risk (odds ratio = 0.90; 95% confidence interval, 0.49-1.65 p=0.74). Similarly no association was seen with rare *TREX1* variants with predicted deleterious effects on enzyme function (odds ratio = 1.05; 95% confidence interval, 0.43-2.61 p=0.91).

***Conclusions:*** No patients with early-onset lacunar stroke had genetic evidence of a *TREX1*-associated monogenic microangiopathy. These results show no evidence of association between rare variants of *TREX1* and early onset lacunar stroke. This includes rare variants that significantly affect protein and enzyme function. Routine sequencing of the *TREX1* gene in patients with early onset lacunar stroke is therefore unlikely to be of diagnostic utility, in the absence of syndromic features or family history.

## Introduction

Cerebral small vessel disease (SVD) causes a quarter of all strokes and is the most common pathology underlying vascular cognitive decline and dementia ^1^. The pathophysiological and genetic basis of SVD is poorly understood, in particular small vessel lacunar stroke ^2, 3^. Rare variants may make a significant contribution to the genetic basis of SVD ^3, 4^ and increasing evidence suggests that monogenic and polygenic forms of SVD share common pathophysiological mechanisms ^5^. For example, dominant missense mutations in *COL4A1* and *COL4A2* cause rare familial forms of cerebral SVD ^6^, and common variants in the same genes are associated with sporadic cerebral small vessel disease ^3^. Such findings demonstrate that genes causing monogenic microangiopathies may also contain variants conferring risk for common forms of cerebral SVD, such as lacunar stroke.

**Figure 1.**
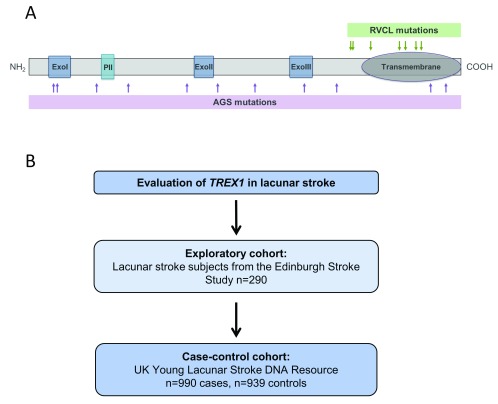
Evaluation of *TREX1* in lacunar stroke. ( **A**) Schematic representation of TREX1 protein, showing known associations with genetic microangiopathic diseases. Shown are monoallelic mutations associated with Retinal Vasculopathy with Cerebral Leukodystrophy (RVCL) and biallelic mutations associated with Aicardi-Goutieres’ Syndrome (AGS). ExoI = exonuclease domain I, ExoII = exonuclease domain II, ExoIII = exonuclease domain III, PII = polyproline domain. ( **B**) Overview of present study. An initial analysis was performed in an exploratory cohort, followed by a case-control study.

TREX1 is a human 3’-5’ exonuclease that can degrade single stranded DNA. Two monogenic small vessel diseases are caused by mutations in *TREX1* ( Figure 1A). Heterozygous frameshift mutations in the C-terminus of *TREX1,* resulting in enzyme mislocalisation, cause retinal vasculopathy with cerebral leukodystrophy (RVCL), an adult-onset systemic microangiopathy with pronounced brain involvement ^7^. Biallelic mutations with loss of enzymatic function can cause Aicardi-Goutières’ Syndrome (AGS), a neonatal onset brain disorder with prominent microangiopathy ^8, 9^ and features of activated innate immunity ^10^. Both genetic cerebral microangiopathies are associated with aberrant innate immune pathways, in particular dysregulation of the type I interferon pathway ^10, 11^. Given the potential for therapeutic modulation of these pathways, evaluation of *TREX1* in SVD phenotypes, such as lacunar stroke, warrants examination. The identification of patients with early-onset cerebral SVD and heterozygous rare *TREX1* variants has led to the hypothesis that such variants might be causally related to early-onset SVD ^12^.

Here we evaluate *TREX1* in patients with small vessel stroke. We perform an initial exploratory analysis in a relatively small cohort of patients with lacunar stroke, and subsequently perform a case-control study in a larger cohort of patients with early-onset lacunar stroke, where small vessel infarction has been confirmed by MRI.

## Methods

### Sanger sequencing

The entire coding sequence of *TREX1* and part of the 5’UTR (-228bp) and 3’ UTR (+57 bp) were amplified by three overlapping amplicons using the following primers:

TREX1 AF: ACAGTCGAATGTGCTGGTCC, TREX1 AR: TCAGACCTGTGATCTCGCTG, TREX1 BF: ACCTCCCACAGTTCCTCCAC, TREX1 BR: TGGTCTCCACTGACAGATGC, TREX1 CF: CTCAGAACACGGCCCAAG, TREX1 CR: ACCACTCAGTGCTATGGGG.

Amplicons were amplified from genomic DNA isolated from peripheral lymphocytes by PCR and checked by agarose gel electrophoresis. Purified PCR products were sequenced in both directions using fluorescent dye terminator chemistry (ABI 3730 DNA analyzer). Sequence reads were analysed by Mutation Surveyor, Sequencher and 4 peaks, and compared to a reference sequence NM_033629 (RefSeq, NCBI).

Rare variants were defined as those with minor allele frequency (MAF) <0.05 (5%) in the Exome Aggregation Consortium ( ExAC) and those absent from ExAC. The presence of all identified variants was confirmed by resequencing.

### Exploratory cohort: Edinburgh Stroke Study

The Edinburgh Stroke Study (Ethics Committee Approval, Lothian Research Ethics Committee LREC/2001/4/46) prospectively recruited consenting ≥18 years old patients with stroke, transient cerebral or monocular ischaemic attack or retinal artery occlusion, admitted to, or seen in outpatient clinics at the Western General Hospital, Edinburgh, between April 2002 and May 2005 ^13^. Stroke was defined as the sudden onset of clinical signs of focal disturbance of cerebral function lasting more than 24 hours with no apparent cause other than that of vascular origin. All patients were of self-reported Caucasian ancestry. Lacunar ischaemic stroke was defined using the Oxfordshire Community Stroke Project (OCSP) lacunar stroke syndrome definition revised in light of site and size of any relevant infarct seen on CT or MRI scan ^14^.

### Case-control cohort: UK Young Lacunar Stroke DNA Resource

The UK Young Lacunar Stroke Study recruited Caucasian patients with lacunar stroke, aged ≤70 years, from 72 specialist stroke services in the UK, between 2002 and 2012 ^15^. The study was approved by the Multi-Centre Research Ethics Committee for Scotland (04/MRE00/36) and written informed consent was obtained from all participants. Lacunar stroke was defined as a clinical lacunar syndrome, with an anatomically compatible lesion on MRI (subcortical infarct ≤15 mm in diameter). All patients underwent full stroke investigation, including brain MRI, imaging of the carotid arteries and ECG. Echocardiography was performed when appropriate. All MRIs and clinical histories were reviewed centrally by one physician (HM). Exclusion criteria were: Any other defined cause, including the following: Stenosis > 50% in the extra- or intracranial cerebral vessels, or previous carotid endarterectomy; cardioembolic source of stroke, defined according to the TOAST (Trial of Org 10172 in Acute Stroke Treatment) criteria ^16^ as high or moderate probability; cortical infarct on MRI; subcortical infarct > 15 mm in diameter, as these can be caused by embolic mechanisms (striatocapsular infarcts); any other specific cause of stroke (e.g. lupus anticoagulant, cerebral vasculitis, dissection, monogenic cause of stroke).

Unrelated Caucasian controls, free of clinical cerebrovascular disease, were obtained by random sampling from general practice lists from the same geographical location as the patients. Sampling was stratified for age and sex. All patients and controls underwent a standardized clinical assessment and completed a standardized study questionnaire. MRI was not performed in controls.

### Variant annotation

Variants were compared with the ExAC database ^17^ to determine a MAF and/or previously identified disease association (ClinVar, NCBI). Variants were sorted by Combined Annotation Dependent Depletion ( CADD). CADD provides a scaled C-score with a C-score of 10 meaning this variant is predicted to be in the top 1% of most deleterious changes in the genome, a score of 20 meaning it is in the top 0.1% ^18^.

### Structural and functional analyses

3D rendering of the variants in a TREX1 dimer ^19^ (Protein DataBase ID: 2OA8, amino acids 5-234) was performed using PyMOL (The PyMOL Molecular Graphics System, Version 1.8 Schrödinger, LLC).

### TREX1-EGFP vector construction

Gateway cloning was used to construct mammalian expression vectors. Briefly, the coding sequence of human *TREX1* was amplified by PCR to include *attB* sites and cloned into pDONR221 (Invitrogen) via BP reaction (BP clonase II kit; Invitrogen). pEGFP-TREX1 was constructed by cloning the *TREX1* coding sequence into a Gateway converted pEGFP-C2 destination vector (Clontech) via LR reaction (LR clonase II kit; Invitrogen). Minipreparations of plasmid DNA (Qiagen) were performed for verification. Midipreparations of plasmid DNA (ZymoResearch) were performed for mammalian cell transfection.

### Site directed mutagenesis

Mutations were introduced into the mammalian expression construct by site-directed mutagenesis, as per manufacturer’s instructions (Q5 Site-Directed Mutagenesis Kit, NEB). Mutations were confirmed by Sanger sequencing.

### TREX1 nuclease activity assay

TREX1 nuclease activity was assayed by transfecting *Trex1* ^-/-^ mouse embryonic fibroblasts (MEFs; a kind gift from Martin Reijns) with pEGFP-TREX1 WT and mutant constructs using Lipofectamine 3000 (Invitrogen). Whole cell protein was extracted with lysis buffer (50 mM Tris, 280 mM NaCl, 0.5% Igepal, 0.2 mM EDTA, 0.2 mM EGTA, 10% glycerol) and protein concentration of the whole cell lysate was determined by Bradford assay (5 X Bradford Reagent, Serva).

Whole cell lysates (final concentration 100 ng/µl) were incubated with nucleic acid substrate (21-mer single stranded oligo with 3’ fluorescein and an internal DABCYL, 3’fl-intDABCYL-TREX1-21mer, sequence TAGACATTGCCCTCG5AGGTAC (Dabcyl dT at position marked 5, 3’ fluorescein); final concentration 200 nM) in reaction buffer (20 mM Tris-HCl, 5 mM MgCl _2_, 2 mM DTT, 100 μg/ml BSA) at room temperature in an opaque 96 well plate. Fluorescent measurements (490 _ex_/525 _em_) were taken with a SpectraMax i3 (Molecular Devices) plate reader over a 90 minute time course with measurements taken every 2 minutes. 

### Statistical analyses

Sequencing, analysis and functional work was performed blind to case-control status. Fisher’s exact test was used to compare proportions of individuals with rare variants in cases versus controls, unless otherwise stated. Odds ratios were calculated using Cochrane RevMan 5. Mann-Whitney U test was used to compare CADD scores between groups. Statistical tests were performed in GraphPad Prism 7.

## Results

### Exploratory cohort: Rare TREX1 variants in the Edinburgh Stroke Study

We first performed an exploratory analysis of patients with lacunar stroke within the Edinburgh Stroke Study ( Figure 1B). This study of >2000 stroke patients includes a subset of 290 patients with a clinical diagnosis of a lacunar stroke. Sanger sequencing of *TREX1* in these 290 patients identified no individuals with genetic results consistent with a diagnosis of RVCL (monoallelic C-terminal frameshift mutations) or AGS (biallelic hypomorphic mutations).

However, four patients with rare heterozygous *TREX1* variants were identified (MAF<0.05, Table 1). The Edinburgh Stroke Study does not include population-matched controls, placing limitation on interpretation of these data. However, compared to published *TREX1* sequencing control data from patients of European ancestry, this is significantly more than would be expected (p = 0.005 Fisher’s exact test) ^20^. Notably, 3 of these patients developed lacunar stroke under the age of 70 years. Recognising that to confirm any potential association between rare *TREX1* variants and lacunar stroke would require more stringent testing, we analysed DNA from a larger independent lacunar stroke cohort with early onset disease (<70 years) together with a population-matched control cohort.

**Table 1.** Rare *TREX1* variants identified in the Edinburgh Stroke Study. Positions of amino acid and nucleotide changes refer to RefSeq assession number NM_033629, the 314 amino acid isoform of TREX1. Combined Annotation Dependent Depletion (CADD) score is a scaled score of predicted pathogenicity. p = 0.005, Fishers Exact Test compared to published control cohort ^20^.

Amino acid alteration	Nucleotide alteration major>minor allele	Total	CADD score	Age
**5' UTR**	-113 A>G	1	0.13	58
**C208S**	623 G>C	1	24	66
**R217R**	651 G>A	1	11.3	85
**E266G**	797 A>G	1	0.05	59

### Case-control study: Rare TREX1 variants in the UK Young Lacunar Stroke Resource

The UK Young Lacunar Stroke Resource (UKYLSR) is a study of approximately 1,000 patients with MRI-confirmed lacunar stroke in patients under the age of 70, with matched population controls. As such this study allows more stringent evaluation of the hypothesis that rare *TREX1* variants confer risk for lacunar stroke. We performed *TREX1* sequencing in cases and controls, including functional annotation and enzymatic assays. We remained blind to case-control status throughout the study.

No individuals in either case or control group had genetic results consistent with a diagnosis of RVCL or AGS.

**Figure 2.**
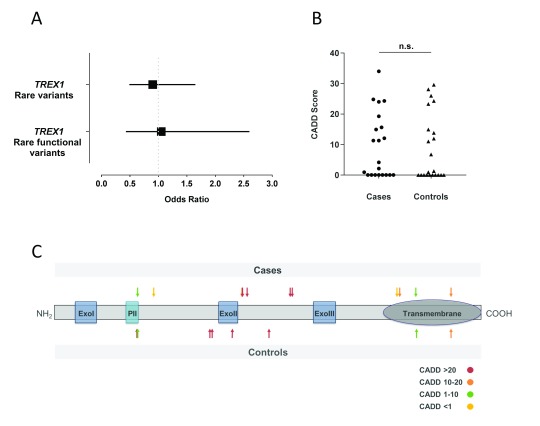
Rare *TREX1* variants in UK Young Lacunar Stroke case and control cohorts. ( **A**) Rare variants in UK Young Lacunar Stroke case and control cohorts. 21 rare heterozygous variants in 990 cases (2.1%) and 22 in 939 controls (2.3%). No association with lacunar stroke was observed, for either rare variants (OR = 0.90; 95% confidence interval, 0.49-1.65 p=0.74) or rare variants with C-scores >10 (OR = 1.05; 95% confidence interval, 0.43-2.6 p>0.99). ( **B**) Distribution of Combined Annotation Dependent Depletion (CADD) scores assigned to rare variants: no significant difference was observed between cases and controls (p=0.72 Mann-Whitney U-test). ( **C**) Schematic representation of TREX1 protein domains showing non-synonymous variants identified in this study in cases or controls (coloured by CADD score).

We next evaluated rare *TREX1* variants (MAF<0.05). We identified 21 rare heterozygous variants in 990 cases (2.1%) and 22 in 939 controls (2.3%). There was no significant association between such variants and lacunar stroke (odds ratio = 0.90; 95% confidence interval, 0.49-1.65 p=0.74; Figure 2A, Table 2). This also held true when only considering non-synonymous variants (16/990 or 1.6% for cases versus 17/939 or 1.8% for controls).

**Table 2.** Rare *TREX1* variants identified in the UK Young Lacunar Stroke Resource. Rare variants in *TREX1* were identified in both cases and controls by Sanger sequencing. Minor allele frequency (MAF; %) of variants that are also present in Exome Aggregation Consortium (ExAC) are shown for comparison. 12/24 variants are novel (not in ExAC). One (P290_A295del) has been reported as a homozygous mutation in AGS1 but is not present in ExAC. The homozygous or compound heterozygous R114H mutation is a common AGS1 mutation (in 14/18 AGS1 families) ^10^. Positions of amino acid and nucleotide changes refer to RefSeq assession number NM_033629, the 314 amino acid isoform of TREX1. CADD, Combined Annotation Dependent Depletion.

Amino acid alteration	Nucleotide alteration major>minor allele	Total	CADD score	ExAC MAF (%)
Cases n=990				
**G2G**	6 C>T	1	15.7	-
**P61P**	183 G>A	1	0.96	0.07
**P73P**	219 G>A	1	4.17	0.002
**A139T**	415 G>A	1	19.3	-
A139Vfs*21	416 GC>G	1	34	0.004
**G142A**	425 G>C	1	24.8	-
**R174G**	520 A>G	1	24	0.0008
**K175N**	525 G>T	1	24.3	-
**R217R**	651 G>A	1	11.3	0.008
**T250T**	750 C>A	1	11.3	-
**A252V**	755 C>A	1	2.15	-
**L254P**	761 T>C	1	12.1	-
**E266G**	797 A>G	8	0.05	0.1691
**P290_A295del**	867 CCCACTGGGTCTGCTGGCC>C	1	15	-
	**Total**	**21**		
Controls n=939				
**P61L**	182 C>T	1	26	0.0008
**P61P**	183 G>A	1	0.96	0.07
**R114H**	341 G>A	1	28.1	0.015
**P116A**	346 C>G	1	23.3	0.0016
**H124H**	372 C>T	1	12	-
**F131I**	391 T>A	1	29.6	-
**A158V**	473 C>T	1	24.3	0.002
**S190S**	570 C>T	1	13.8	-
**E266G**	797 A>G	10	0.05	0.1691
**G286E**	857 G>A	1	11	-
**P290_A295del**	867 CCCACTGGGTCTGCTGGCC>C	1	15	-
**3' UTR**	+17 T>C	1	6.78	-
**3' UTR**	+37 T>C	1	1.33	1.23
	**Total**	**22**		

**Figure 3.**
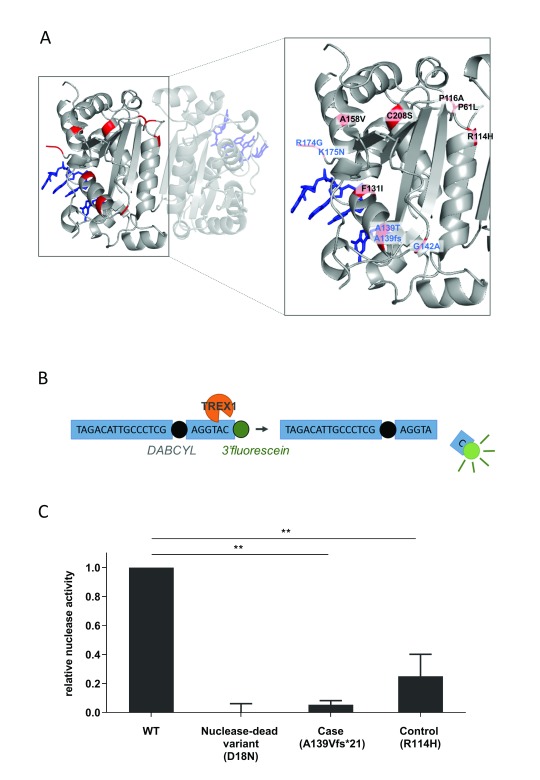
Rare variants can affect TREX1 nuclease activity. ( **A**) Location of rare variants (CADD>10) mapped onto TREX1 dimer structure (highlighted in red), with coordinates taken for mouse TREX1 in complex with ssDNA (blue). Variants in the case cohort are highlighted in blue. ( **B**) TREX1 nuclease assay measures nuclease activity as release of 3’fluorescein from an oligonucleotide containing a DABCYL quencher. ( **C**) Relative nuclease activity of predicted most severe variants identified from case (A139Vfs*21) and control groups (R114H) of the UK Young Lacunar Stroke Study. Nuclease activity was assayed in total protein supernatants from *Trex1 ^-/-^* MEFs transfected with TREX1 expression constructs containing variants generated by site directed mutagenesis. Rare variants identified in the Young Lacunar stroke cohorts were compared with a known nuclease-dead variant (D18N). Data shown is average of two or more independent experiments performed in triplicate ± standard deviation of the independent experiments relative to WT ** p<0.01.

Variants differ in their capacity to reduce enzymatic function of TREX1. For example some mutations such as D18N can cause complete loss of function of exonuclease activity ^7^. We therefore next considered annotations of these variants which evaluated the potential pathogenicity of a given variant. CADD is a method for integrating diverse functional annotations into a single measure (CADD score, or C-score), which can predict the potential pathogenicity of a variant *in silico* ^18^. When rare variants with low CADD scores (<10) were excluded, functional rare variants were identified at a frequency of 10/990 in cases (1.0%) and 9/939 (0.96%) in controls (OR 1.05; 95% confidence interval, 0.43-2.61 p=0.91, Figure 2B). The CADD scores for rare variants did not differ significantly between groups ( Figure 2B, p=0.72 Mann-Whitney U test). The location of variants within *TREX1* influences clinical phenotype in monogenic microangiopathic disease ( Figure 1A). The variants we identified were distributed throughout the *TREX1* gene, and there was no apparent spatial clustering when variants were mapped onto a 3D protein model ( Figure 2C, Figure 3A).

### Rare TREX1 variants can decrease exonuclease activity

To confirm that rare variants with high CADD scores exert a deleterious effect on protein function, we evaluated the effect of rare variants on TREX1 exonuclease activity with a high C-score from each group. We identified a variant from each group with a CADD score >20 and thus predicted to confer significant pathogenic effect on the protein. To examine such amino acid changes on TREX1 function, we reconstituted mouse *Trex1* ^-/-^ MEFs with the mutated allele, generated by site-directed mutagenesis and assessed cellular nuclease activity against a ssDNA substrate ( Figure 3B). While wildtype TREX1 reconstituted nuclease activity against ssDNA, rare variants from both groups (Case: A139Vfs*21, C-score 34. Control: R114H, C-score 28) lead to significant loss of 3’-5’ exonuclease activity ( Figure 3C).

Together these results show no evidence for an association between rare variants of *TREX1* and early onset lacunar stroke, including variants that exert deleterious effects on protein function.

## Discussion

There is a need to identify aetiological factors in small vessel stroke, in particular molecular pathways that might be amenable to therapeutic intervention ^1^. Recent meta-analyses of GWAS studies have suggested that the “missing heritability” of small vessel stroke may be in part attributed to rare variants ^2, 21^. One possibility is that mutations in genes that cause monogenic small vessel diseases, such as *NOTCH3*, *HTRA1, COL4A1* and *TREX1*, might confer risk for sporadic lacunar stroke. This hypothesis is strengthened by the identification of an association between sporadic SVD phenotypes and common variants in *COL4A1/2,* since mutations in these genes can cause monogenic SVD ^3^.

*TREX1* is therefore an important candidate gene to evaluate in lacunar stroke. Biallelic and monoallelic mutations in *TREX1* can cause two clinically distinct monogenic syndromes characterized by prominent microangiopathy, AGS and RVCL. While the molecular events by which altered TREX1 function causes SVD is unknown, increasing lines of evidence suggest an association with activated innate immunity, including pathways that are potentially amenable to therapeutic intervention ^11, 22^. As such detailed evaluation of this gene in sporadic small vessel stroke phenotypes is a priority. 

Here we test the hypothesis that rare variants of *TREX1* are associated with lacunar stroke, in particular early onset disease. We first examine DNA from an exploratory cohort, recognizing important limitations in the interpretation of genetic data from small uncontrolled studies. Consistent with other screening studies of this gene in other early-onset SVD phenotypes ^12^, we identified rare variants of *TREX1* in about 1.3% of cases, and observed that 3 out of 4 of these cases were under 70 years of age. Comparison of this proportion to published control data suggested a potential association of early onset lacunar stroke with rare variants of *TREX1*. However, such analyses present serious methodological limitations, since published control cohorts are not matched for geographical region and age. Therefore, although this type of comparison might be useful in generating preliminary data on which to focus and power more detailed studies, the statistical analysis of this preliminary cohort is prone to bias, confounding and chance ^23^.

Therefore to assess whether our observations in this preliminary cohort represented a real association, we performed a more methodologically rigorous evaluation of *TREX1* in the UKYLSR. This differs from the exploratory study in a number of ways, which allow more robust genetic conclusions to be drawn. Firstly, the UKYLSR is a dedicated study of early onset lacunar stroke. Secondly, inclusion in the study requires confirmation of a small vessel stroke by MRI. This is important since a clinical diagnosis of a lacunar syndrome may not necessarily be caused by a small vessel stroke ^1^. Thirdly, the study was controlled with an age, sex and geographically matched control population. We remained blinded to case-control status throughout the study, including analyses of the functional consequences of rare variants.

The results of this case-control study showed no evidence that rare variants in *TREX1* are associated with small vessel stroke. In the UKYLSR, rare variants in *TREX1* occur in about 2% of both cases and controls. As such rare variants occur more frequently than previously detected in different control populations, highlighting potential population variation and reinforcing the need for dedicated age and population-matched control cohorts ^20^. Our findings emphasize the importance of confirmation cohorts in genetic association studies, however persuasive the prior biological rationale ^24^.

These rare variants included those that can directly alter protein structure and function. The distribution of CADD scores, which reflect an *in silico* evaluation of the potential pathogenicity of variants, was not different between groups. We show that rare variants with high CADD scores, which can affect enzymatic function *in vitro,* are observed at similar frequencies in both cases and control populations.

Our results are consistent with a recently published next-generation sequencing study comparing approximately 600 lacunar stroke patients with control individuals from the INTERSTROKE cohort ^25^. This study showed that rare variants in monogenic stroke genes, including *TREX1,* were not associated with lacunar stroke phenotypes. A potential limitation of both studies is lack of statistical power, although unbiased publication of such sequencing studies will allow meta-analyses with higher degrees of power to be performed.

These results have implications relevant for clinical practice. Firstly, none of the 1,280 lacunar stroke patients sequenced here had genetic results consistent with monogenic *TREX1*-associated genetic microangiopathies. Secondly, the identification of rare heterozygous variants of *TREX1* in early onset small vessel stroke, even those that confer substantial functional effects, may not be of clinical relevance, although our analysis does not exclude a weak effect. Taken together, these findings do not support routine testing of *TREX1* variants in early onset small vessel stroke, in the absence of syndromic features or a supportive family history. Furthermore, the interpretation of rare *TREX1* variants in early onset SVD phenotypes obtained through screening ^12^ or next generation sequencing approaches ^25^, should be interpreted with caution given that they are observed in control populations at a frequency of approximately 2%.

## Data availability

The data referenced by this article are under copyright with the following copyright statement: Copyright: © 2017 McGlasson S et al.

Raw data for this study are available from OSF: http://doi.org/10.17605/OSF.IO/2K4AP ^26^
