# Supplementary material for: Rare variants of the 3’-5’ DNA exonuclease TREX1 in early onset small vessel stroke
Source: Wellcome Open Res. 2017 Nov 2;2:106. [Version 1] doi: 10.12688/wellcomeopenres.12631.1 (PMC5717473; doi:10.12688/wellcomeopenres.12631.1)
Supplement: Peer review history

# Peer review: Referee response for version 1

**DOI:** 10.21956/wellcomeopenres.13676.r27573

**Published:** 2017-12-04

**Authors:** Arne Lindgren

**Affiliations:** 1 Department of Clinical Sciences Lund, Neurology, Lund University, Lund, Sweden

This is a study of 290 subjects with lacunar stroke from the Edinburgh Stroke Study and 990 subjects with lacunar stroke plus 939 control subjects from the UK Lacunar Stroke Resource. The authors examined if there was an association between variants in *TREX1* and lacunar stroke but could not detect this. This is an important and relevant finding because when taking previous reports into consideration it has been reasonable to suspect a relation between *TREX1* variants and lacunar stroke. Some comments:

 Microangiopathy can cause many different phenotypes and a discussion about how closely the different microangiopathies noted in previous studies of *TREX1* can be supposed to be related to the phenotype lacunar stroke would be of interest. Maybe the reported microangiopathies should instead be suspected to be related to other phenotypes than lacunar stroke? It would therefore be valuable if the authors, with their acknowledged expertise, in the manuscript could explain more about the possible relationships between microangiopathy and SVD causing lacunar stroke.

 For clarity of the manuscript, the authors should consider moving some parts of the Results section to the Methods section. This includes the paragraph beginning with “The UK Young Lacunar Stroke Resource (UKYLSR) is a study of approximately 1,000 patients” and the sentences starting with: “We therefore next considered annotations of these variants which evaluated the potential pathogenicity of a given variant. CADD is a method…”

 The authors also used mouse *TREX1* for their evaluations. It would be valuable if they could comment in e.g. their Discussion whether any differences between mouse *TREX1* and human *TREX1* may be of importance.

 Suggest that the authors omit the sentence “p = 0.005, Fishers Exact Test compared to published control cohort” from the legend of Table 1. This is already more clearly explained in the text on page 5.

 The abbreviation Exo is explained in Figure 1 but not in Figure 2.

I have read this submission. I believe that I have an appropriate level of expertise to confirm that it is of an acceptable scientific standard.

# Peer review: Referee response for version 1

**DOI:** 10.21956/wellcomeopenres.13676.r27572

**Published:** 2017-11-13

**Authors:** John P. Atkinson, Andria L. Ford

**Affiliations:** 1 Division of Rheumatology, Washington University School of Medicine, St. Louis, MO, USA; 2 Department of Neurology, Washington University School of Medicine, Saint Louis, MO, USA

I recommend this paper be published as it has important albeit largely negative data. There are several points though that the authors should consider addressing.

 Authors:

1. The authors are to be commended on putting together their remarkable cohorts – numbers are impressive. Several reports of “associations” such as in Reference 20 are under powered and the authors nicely point this out.
2. Another point to make is that RVCL mutations are frame-shift. The protein these patients produce is lacking its COOH-tail. TREX1 is on the “loose” so to speak. The variants identified herein in the COOH tail in stroke or control cohorts herein would thus not be expected to lead to RVCL.
3. The authors should comment that testing for the presence of lacunar stroke is not the same as testing for accelerated small vessel disease. We don’t see lacunar strokes in patients with RVCL/CRV/TREX1 vasculopathy. The ischemic damage is even on a smaller scale (truly a microangiopathy).
4. A medical biostatistician specially in genetics should review this paper.

We have read this submission. We believe that we have an appropriate level of expertise to confirm that it is of an acceptable scientific standard.
